# Computational model of interictal discharges triggered by interneurons

**DOI:** 10.1371/journal.pone.0185752

**Published:** 2017-10-04

**Authors:** Anton V. Chizhov, Dmitry V. Amakhin, Aleksey V. Zaitsev

**Affiliations:** 1 Computational Physics Laboratory, Ioffe Institute, Saint Petersburg, Russia; 2 Laboratory of Molecular Mechanisms of Neural Interactions, Sechenov Institute of Evolutionary Physiology and Biochemistry of the Russian Academy of Sciences, Saint Petersburg, Russia; 3 Institute of Experimental Medicine, Federal Almazov North-West Medical Research Centre, Saint Petersburg, Russia; Georgia State University, UNITED STATES

## Abstract

Interictal discharges (IIDs) are abnormal waveforms registered in the periods before or between seizures. IIDs that are initiated by GABAergic interneurons have not been mathematically modeled yet. In the present study, a mathematical model that describes the mechanisms of these discharges is proposed. The model is based on the experimental recordings of IIDs in pyramidal neurons of the rat entorhinal cortex and estimations of synaptic conductances during IIDs. IIDs were induced in cortico-hippocampal slices by applying an extracellular solution with 4-aminopyridine, high potassium, and low magnesium concentrations. Two different types of IIDs initiated by interneurons were observed. The first type of IID (IID1) was pure GABAergic. The second type of IID (IID2) was induced by GABAergic excitation and maintained by recurrent interactions of both GABA- and glutamatergic neuronal populations. The model employed the conductance-based refractory density (CBRD) approach, which accurately approximates the firing rate of a population of similar Hodgkin-Huxley-like neurons. The model of coupled excitatory and inhibitory populations includes AMPA, NMDA, and GABA-receptor-mediated synapses and gap junctions. These neurons receive both arbitrary deterministic input and individual colored Gaussian noise. Both types of IIDs were successfully reproduced in the model by setting two different depolarized levels for GABA-mediated current reversal potential. It was revealed that short-term synaptic depression is a crucial factor in ceasing each of the discharges, and it also determines their durations and frequencies.

## 1 Introduction

Temporal lobe epilepsy is characterized by the presence of several types of abnormal electrical activity within the brain, which are most commonly referred to as ictal and interictal discharges. Interictal discharges (IIDs) typically occur in between seizure-like events; however, the first IIDs precede ictal activity and increase in frequency during the development of the disease [1]. IIDs have been studied both experimentally and by mathematical modeling [2], [3], [4], [5], [6], [7], [8], [9]; however, the mechanisms of IIDs are not entirely understood. Depending on the experimental model used, different types of IIDs have been observed and different mechanisms of IIDs have been proposed.

Understanding the mechanisms requires answering the questions regarding which process initiates each discharge, which types of neurons contribute to the discharge generation, and why the high firing activity that begins at the onset of IIDs terminates. Certain kind of interictal and preictal discharges are based on the recurrent interactions of only excitatory neurons [2], [10] whereas the mechanisms of other discharges have suggested that interneurons are strongly involved [11], [10], [4], [12], [13]. Using 4-aminopyridine (4-AP) in vitro epilepsy model [14], we distinguished two basic types of IIDs: IID1s, which are mediated by GABA, and IID2s, which are mediated by both GABA and glutamate. The depolarizing effect of GABA may cause the generation of epileptiform events. It is known that some convulsants trigger an intense firing of interneurons, which enhances GABA release [15] increases intracellular chloride concentration and may switch GABA responses to depolarization [16], [17], [18], [13]. In our previous work, a synchronous interneuronal activity with IIDs has been described, that evoked by using 4-AP, high potassium in vitro model [14]. Similar events were referred to as GABA-mediated, slow interictal potentials by [19]. Such events were recorded by Yekhlef et al. [12] in rat entorhinal cortex slices in a 4-AP-containing solution and by Pallud et al. [13] in slices of human neocortex with glioma infiltration. Dickson and Alonso [20] obtained pure GABA-mediated events evoked in slices by carbachol. As shown by Michelson and Wong [21], GABAergic interneurons may become synchronized via (1) recurrent interneuron collaterals and the depolarizing action of synaptically activated GABAA receptors and (2) electrotonic coupling. In the present paper, based on the experimental data, a mathematical model that implies an interaction between glutamatergic and GABAergic neuronal populations during IIDs triggered by a spontaneous GABA release followed by a recurrent GABAergic excitation of the interneurons is proposed.

Determining which mechanism is involved in discharge termination is the next key question regarding IIDs. Some models suggest a primary role of AHP-like channels in the decrease of firing during a single discharge [3]; however, the firing frequency adaptation is not prominent for a significant fraction of interneurons [22], [23], [24]. Therefore, the termination of interneuron-based IIDs cannot be fully explained based on the role of these channels. Another factor is short-term synaptic depression [25], which was also used in a modeling study of epileptic discharges [26]. The crucial role of short-term synaptic depression in the modulation of the probability and duration of the synchronous discharges of the CA3 network has been shown in an experimental study by Staley et al. [2], in which the depletion of the releasable glutamate pool at recurrent synapses was revealed. Still, its role in the termination of other types of IIDs has not been revealed. In the current model, synaptic depression has been suggested as one of the key elements of the IID mechanism.

The proposed mathematical model is grounded on recent whole-cell patch-clamp recordings in combined hippocampal-entorhinal cortex slices with high potassium/low magnesium concentrations and 50 *μ*M 4-AP in an extracellular solution [14], which were analyzed by means of synaptic conductance estimations. According to the findings, there are two different types of IIDs. IIDs of the first type (IID1) reflect synchronization in the network of interneurons; thus, they are characterized by pure GABAergic currents recorded in an excitatory neuron. IIDs of the second type (IID2) are composed of both GABAergic and glutamatergic components. Spontaneous GABAergic activity triggers a cascade of recurrent GABAergic and glutamatergic excitation. Thus, GABA-conductance is the first component that contributes to IID2. Then, AMPA-conductance rapidly increases, followed by NMDA-conductance. To develop a mathematical model that was consistent with the experimental data, a population approach was used, namely the conductance-based refractory-density (CBRD) model [27]. This method provides both a biophysically detailed description of neuronal populations regarding ionic channel conductances for one- or two-compartment neurons and good precision for statistically equilibrium and non-equilibrium regimes of ensemble activities. Hence, it combines the advantages of the alternative approaches applied to epilepsy, which are direct network modeling and mean-field models, reviewed by Wendling et al. [7]. The model describes IIDs as a network activity of recurrently connected excitatory and inhibitory populations [28], which is controlled by a shift of the reversal potential of GABA-mediated currents and strongly depends on the effects of synaptic depression.

## 2 Materials and methods

### 2.1 Experiments

Details of the experimental methods were described previously [14]. Shortly, the experiments were carried out in 3-week-old Wistar rats. All animal procedures followed the guidelines of the European Community Council Directive 86/609/EEC and were approved by the Animal Care and Use Committee of the Sechenov Institute of Evolutionary Physiology and Biochemistry of the Russian Academy of Sciences. Rats were decapitated and their brains removed rapidly. A vibrating microtome (Microm HM 650 V; Microm; Germany) was used to cut horizontal slices 300-μm thick that contained entorhinal cortex and hippocampus. All steps used artificial cerebrospinal fluid (ACSF) with the following composition (in mM): 126 NaCl, 2.5 KCl, 1.25 NaH_2_PO_4_, 1 MgSO_4_, 2 CaCl_2_, 24 NaHCO_3_, and 10 dextrose. The ACSF was aerated with carbogen (95% O2/5% CO_2_). Recordings were made at 30°C. Pyramidal neurons in deep layers of the entorhinal cortex were visualized using a Zeiss Axioscop 2 microscope (Zeiss; Oberkochen, Germany) equipped with differential interference contrast optics and a video camera (PointGrey Grasshopper3 GS3-U3-23S6M-C, FLIR Integrated Imaging Solutions Inc., USA). Patch electrodes (3–5 MΩ) were pulled from borosilicate filamented glass capillaries (WPI; UK) on a P-1000 Micropipette Puller (Sutter Instrument; Novato, CA, USA). For current-clamp recordings, a potassium-gluconate-based filling solution (iS-1) was used. iS-1 had the following composition (in mM): 135 K-gluconate, 10 NaCl, 5 EGTA, 10 HEPES, 4 ATP-Mg, and 0.3 GTP (with pH adjusted to 7.25 with KOH). For voltage-clamp recordings, a solution based on cesium-methane-sulfonate (CsMeS) (iS-2) was used. iS-2 had the following composition (in mM): 127 CsMeS, 10 NaCl, 5 EGTA, 10 HEPES, 6 QX314, 4 ATP-Mg, and 0.3 GTP (with pH adjusted to 7.25 with CsOH). Whole-cell recordings were performed with two Model 2400 patch-clamp amplifiers(AM-Systems; Sequim, WA, USA), and an NI USB-6343A/D converter (National Instruments; Austin, TX, USA) using WinWCP5 software (SIPBS; Glasgow, UK). The data were filtered at 10 kHz and sampled at 20 kHz. After formation of the whole-cell configuration, access resistance was less than 15 MΩ and remained stable (≤ 30% increase) during the experiments in all cells included.

In the combined entorhinal cortex-hippocampal slices, epileptiform activity was induced with the pro-epileptic solution (eS), containing the following (in mM): 120 NaCl, 8.5 KCl, 1.25 NaH_2_PO_4_, 0.25 MgSO_4_, 2 CaCl_2_, 24 NaHCO_3_, 10 dextrose, and 0.05 4-AP. The solution was aerated with carbogen (95% O2/5% CO2) throughout the experiment. The flow rate in the perfusion chamber was 5–6 ml/min. The liquid junction potentials were measured as described [29], and the holding potential was compensated offline for voltage-clamp recordings by subtracting 7 mV. To evoke synaptic responses the stimulating electrode was placed in the same layer of the entorhinal cortex as the recorded neuron at a distance of 100–200 μm. We estimated synaptic conductance as described previously [14]. Shortly, we registered IID1 and IID2 in voltage-clamp mode at various levels of membrane potential. Next, we averaged 5–25 of the recorded IID1s or IID2s. From each set of currents the I-V curves were formed every 1 ms, which were fitted with a three-parameter function

$$I_{total}\left(V;g_{AMPA},g_{GABA},g_{NMDA}\right)=g_{AMPA}\;f_{AMPA}\left(V\right)+g_{GABA}\;f_{GABA}\left(V\right)+g_{NMDA}\left(V\right)\;f_{NMDA}\left(V\right)$$

This function is a weighted sum of pre-recorded I-V relationships of AMPAR-, NMDAR-, and GABAR-mediated currents *f*_*AMPA*_(*V*), *f*_*GABA*_(*V*) and *f*_*NMDA*_(*V*) with corresponding synaptic conductances serving as coefficients.

### 2.2 Modeling: CBRD-approach for a population of pyramidal neurons

The model of synaptically interacting neuronal populations is based on our previous study [28]. We consider excitatory and inhibitory neuronal populations, denoted by indexes *E* and *I*, correspondingly. The types of synapses are denoted by the types of mediator and postsynaptic neurons as follows: (*AMPA*, *j*), (*GABA*, *j*) and (*NMDA*, *j*) with the postsynaptic index *j* = *E* or *I*.

A mathematical description of each single population is based on the probability density approach, namely, CBRD approach [27]. The CBRD approach provides both a biophysically detailed description of a single neuronal population in terms of ionic channel conductances for one- or two-compartment neurons and good precision for statistically equilibrium and non-equilibrium regimes of ensemble activity. Thus, the approach takes advantages of both neural network simulations and population models. Known neuronal population models are either very simplified models such as firing-rate or neural mass, or are based on the probability-density approach. The simplified models are not entirely adequate for transient process simulations because of their underlying assumption that neurons are always desynchronized. In contrast, the probability density approach avoids this assumption; however, this method is commonly applied only to simplified, one-variable neurons like integrate-and-fire. This limitation has been overcome in our works that derive a CBRD applicable to regular-spiking, adaptive, and fast-spiking neurons described in terms of Hodgkin-Huxley (HH)-like approximations [27], [30]. This model is quite efficient for simulations of all-to-all coupled populations [28]. The CBRD approach has been recently validated with experiments and generalized to the case of lognormal weights in [31].

The approach considers a population of an infinite number of Hodgkin-Huxley-like neurons receiving both a common input and an individual for each neuron noise. In any arbitrary case of transient or steady-state stimulation the firing rate of such population can be quite precisely and computationally efficiently calculated by solving a system of equations in partial derivatives, 1-d transport equations. The equations govern an evolution of neuronal states distributed in a phase space of the time elapsed since last spikes, t*. They contain the Hodgkin-Huxley equations for the membrane voltage and gating variables, parameterized by t*, as well as the equation for the neuronal density in t*-space, *ρ*^*E*^(*t*, t*). The output characteristic of the population’s activity is the firing rate *ν*^*E*^(*t*), which is equal to *ρ*^*E*^ in the state of a spike, t* = 0. The equations written below describe an excitatory population of adaptive regular spiking pyramidal cells according to [27] and [30].

Basic neurons have 2-compartments, according to [32], with the somatic and dendritic voltages *U*^*E*^(*t*, t*) and $U_{d}^{E}\left(t,t^{*}\right)$. In comparison with the one-compartment model, the extra parameters is the ratio of dendritic to somatic conductances *γ* and the dendritic length. We assume that the inhibitory synapses are located at soma, contributing into the somatic synaptic current *I*_*soma*_, whereas the excitatory synapses are at dendrites, determining the dendritic synaptic current *I*_*dendr*_. In spite of different locations of the synapses, both excitatory and inhibitory conductances are attributed to the somatic compartment, to be compared with experimental whole-cell somatic registrations. For that purpose, the model from [32] is constructed such that it implicitly solves a reverse voltage-clamp problem and thus estimates the dendritic synaptic current. The implementation of the 2-compartment model in the CBRD-model is straightforward, it increases the number of equations but not the number of independent variables.

Approximations of voltage-gated ionic currents used here and in [27] are based on the CA1 pyramidal cell model from [33], where instead of full description of calcium dynamics and calcium-dependent potassium currents a cumulative after-spike hyperpolarization (AHP) current was introduced according to [34], that provides an effect of slow spike timing adaptation. Parameterized by t*, the governing equations are as follows:

$$\begin{array}{c} \frac{\partial \rho^{E}}{\partial t}+\frac{\partial \rho^{E}}{\partial t^{*}}=-\rho^{E}H\left(U^{E},g_{tot}^{E}\right), \end{array}$$ (1)

$$C\;(\frac{\partial U^{E}}{\partial t}+\frac{\partial U^{E}}{\partial t^{*}})=-g_{L}\left(U^{E}-V_{rest}\right)+\frac{2\gamma }{l}\;g_{L}\left(U_{d}^{E}-U^{E}\right)\;-I_{Dr}-I_{A}-I_{M}-I_{AHP}+I_{soma}$$ (2)

$$\begin{array}{c} C\;(\frac{\partial U_{d}^{E}}{\partial t}+\frac{\partial U_{d}^{E}}{\partial t^{*}})=-g_{L}\left(U_{d}^{E}-V_{rest}\right)-\frac{2}{l}\;g_{L}\left(U_{d}^{E}-U^{E}\right)+\frac{I_{dendr}}{\gamma }, \end{array}$$ (3)

where $g_{tot}^{E}\left(t,t^{*}\right)$ is the total conductance; *l* is the square ratio of the dendritic length to the characteristic length. The somatic and dendritic synaptic currents *I*_*soma*_ and *I*_*dendr*_ are calculated as

$$\begin{array}{c} I_{soma}=g_{GABA,E}\left(t\right)\left(V_{GABA}-U^{E}\right) \end{array}$$

$$\begin{array}{ccc} I_{dendr} & = & \left(\frac{l\;\tau_{m}^{0}}{2}\frac{d}{dt}+1+\frac{l}{2}\right)\\ & & (g_{AMPA,E}\left(t\right)\left(V_{AMPA}-U^{E}\right)+g_{NMDA,E}\left(t\right)\left(V_{NMDA}-U^{E}\right)), \end{array}$$

where the differential operator represents the solution of the reverse problem of dendritic current estimation from somatically registered-like conductances [32]. We imply that the synaptic conductance kinetics is estimated from somatic responses to stimulation of presynaptic neuronal population. Thus it implicitly accounts not only the kinetics of synaptic channels but also the dendritic and axonal propagation delays. For the dendritic compartment, the differential operator sharpens the transient effect of the channels, thus providing better agreement between somatic postsynaptic currents and potentials. Note that we take into account this sharpening only for glutamatergic channels by placing them in the dendritic compartment [28].

#### Hazard function

The source term in the Eq (1) is the hazard function *H* which is defined as the probability for a single neuron to generate a spike, if known actual neuron state variables. The hazard function *H* has been approximated in [27] for the case of white noise and in [30] for the case of color noise as a function of *U*(*t*) and *s*(*t*), and parameters *σ*, *V*_*th*_ and the ratio of membrane to noise time constants *k* = *τ*_*m*_/*τ*_*Noise*_:

$$\begin{array}{c} H(U)=A+B, \end{array}$$ (4)

$$\begin{array}{c} A=\frac{1}{\tau_{m}}e^{0.0061-1.12\;T-0.257\;T^{2}-0.072\;T^{3}-0.0117\;T^{4}}\;(1-{\left(1+k\right)}^{-0.71+0.0825(T+3)}), \end{array}$$

$$\begin{array}{c} B=-\sqrt{2}\;{\left[\frac{dT}{dt}\right]}_{+}\tilde{F}\left(T\right),\;\;\;\tilde{F}\left(T\right)=\sqrt{\frac{2}{\pi }}\;\frac{\exp(-T^{2})}{1+\text{erf}(T)},\;\;\;T=\frac{V_{th}-U}{\sqrt{2}\;\sigma_{V}}, \end{array}$$

where *T* is the membrane potential relative to the threshold, scaled by noise amplitude; *A* is the hazard for a neuron to cross the threshold because of noise, derived analytically in [27] and approximated by exponential and polynomial for convenience; *B* is the hazard for a neuron to fire because of depolarization due to deterministic drive, i.e. the hazard due to drift in the voltage phase space. Note that the *H*-function is independent of the basic neuron model and does not contain any free parameters or functions for fitting to any particular case. Thus, *H*-function is the same for excitatory and inhibitory populations.

#### Ionic voltage-dependent channels

In our previous works [27] and [28], approximations for ionic currents were taken from [33] with some reductions related to calcium channels. The set of currents includes the voltage-dependent potassium currents *I*_*DR*_ and *I*_*A*_ responsible for spike repolarization, the slow potassium current *I*_*M*_ that contributes to spike frequency adaptation, and the potassium current *I*_*AHP*_, implicitly dependent on calcium dynamics, which also contributes to spike frequency adaptation. The approximating formulas for the currents *I*_*DR*_, *I*_*A*_ and *I*_*M*_ are taken from [33]; the approximation for *I*_*AHP*_ is from [34]. In conventional notations for maximum conductances ${\bar{g}}_{\ldots }$, reversal potentials *V*_…_, activation and inactivation variables *x* and *y*, the approximations parameterized by t* are following.

The voltage-dependent potassium current *I*_*DR*_:

$$\begin{array}{ccc} I_{DR}\left(U^{E},t,t^{*}\right) & = & {\bar{g}}_{DR}\;x\left(t\right)\;y\left(t\right)\;\left(U^{E}\left(t\right)-V_{K}\right), \end{array}$$ (5)

$$\begin{array}{c} \frac{\partial x}{\partial t}+\frac{\partial x}{\partial t^{*}}=\frac{x_{\infty }\left(U^{E}\right)-x}{\tau_{x}\left(U^{E}\right)}, \end{array}$$ (6)

$$\begin{array}{c} \frac{\partial y}{\partial t}+\frac{\partial y}{\partial t^{*}}=\frac{y_{\infty }\left(U^{E}\right)-y}{\tau_{y}\left(U^{E}\right)} \end{array}$$ (7)

$$\begin{array}{ccc} & & \tau_{x}=1/\left(a+b\right)+0.8\;\text{ms};\\ & & x_{\infty }=a/\left(a+b\right),\\ & & a=0.17\exp\left(\left(U^{E}+5\right)\cdot 0.090\right)\;{\text{ms}}^{-1},\\ & & b=0.17\exp\left(-\left(U^{E}+5\right)\cdot 0.022\right)\;{\text{ms}}^{-1},\\ & & \tau_{y}=300\;\text{ms},\\ & & y_{\infty }=1/\left(1+\exp\left(\left(U^{E}+68\right)\cdot 0.038\right)\right); \end{array}$$

The voltage-dependent potassium current *I*_*A*_:

$$\begin{array}{c} I_{A}\left(U^{E},t,t^{*}\right)={\bar{g}}_{A}\;x^{4}\left(t\right)\;y^{3}\left(t\right)\;\left(U^{E}\left(t\right)-V_{K}\right), \end{array}$$ (8)

$$\begin{array}{c} \frac{\partial x}{\partial t}+\frac{\partial x}{\partial t^{*}}=\frac{x_{\infty }\left(U^{E}\right)-x}{\tau_{x}\left(U^{E}\right)}, \end{array}$$ (9)

$$\begin{array}{ccc} \frac{\partial y}{\partial t} & + & \frac{\partial y}{\partial t^{*}}=\frac{y_{\infty }\left(U^{E}\right)-y}{\tau_{y}\left(U^{E}\right)} \end{array}$$ (10)

$$\begin{array}{ccc} & & \tau_{x}=1/\left(a_{x}+b_{x}\right)+1\;\text{ms};\\ & & x_{\infty }=a_{x}/\left(a_{x}+b_{x}\right),\\ & & a_{x}=0.08\exp\left(\left(U^{E}+41\right)\cdot 0.089\right)\;{\text{ms}}^{-1},\\ & & b_{x}=0.08\exp\left(-\left(U^{E}+41\right)\cdot 0.016\right)\;{\text{ms}}^{-1},\\ & & \tau_{y}=1/\left(a_{y}+b_{y}\right)+2\;\text{ms};\\ & & y_{\infty }=a_{y}/\left(a_{y}+b_{y}\right),\\ & & a_{y}=0.04\cdot \exp\left(-\left(U^{E}+49\right)\cdot 0.11\right)\;{\text{ms}}^{-1},\\ & & b_{y}=0.04\;{\text{ms}}^{-1}; \end{array}$$

The voltage-dependent potassium current *I*_*M*_:

$$\begin{array}{c} I_{M}\left(U^{E},t,t^{*}\right)={\bar{g}}_{M}\;x^{2}\left(t\right)\;\left(U^{E}\left(t\right)-V_{M}\right), \end{array}$$ (11)

$$\begin{array}{c} \frac{\partial x}{\partial t}+\frac{\partial x}{\partial t^{*}}=\frac{x_{\infty }\left(U^{E}\right)-x}{\tau_{x}\left(U^{E}\right)}, \end{array}$$ (12)

$$\begin{array}{ccc} & & \tau_{x}=1/\left(a+b\right)+8\;\text{ms},\\ & & x_{\infty }=a/\left(a+b\right),\\ & & a=0.003\exp\left(\left(U^{E}+45\right)\cdot 0.135\right)\;{\text{ms}}^{-1},\\ & & b=0.003\exp\left(-\left(U^{E}+45\right)\cdot 0.090\right)\;{\text{ms}}^{-1}; \end{array}$$

The adaptation current *I*_*AHP*_:

$$\begin{array}{c} I_{AHP}\left(U^{E},t,t^{*}\right)={\bar{g}}_{AHP}\;w\left(t\right)\;\left(U^{E}\left(t\right)-V_{K}\right), \end{array}$$ (13)

$$\begin{array}{c} \frac{\partial w}{\partial t}+\frac{\partial w}{\partial t^{*}}=\frac{w_{\infty }\left(U^{E}\right)-w}{\tau_{w}\left(U^{E}\right)}, \end{array}$$ (14)

$$\begin{array}{ccc} & & \tau_{w}=2000/(3.3\exp\left(\left(U^{E}+35\right)/20\right)\;+\exp(-\left(U^{E}+35\right)/20))\;\text{ms},\\ & & w_{\infty }=1/\left(1+\exp\left(-\left(U^{E}+35\right)/10\right)\right); \end{array}$$

#### Boundary conditions

According to the conservation of the number of neurons in a population, the firing rate is calculated as a sink of neurons from their state t* due to spiking, *ρ*^*E*^(*t*, t*) *H*(*U*^*E*^(*t*, t*), integrated over the whole phase space, i.e.

$$\begin{array}{c} \nu^{E}\left(t\right)\equiv \rho^{E}\left(t,0\right)=\int_{+0}^{\infty }\;\rho^{E}\left(t,t^{*}\right)\;H\left(U^{E}\left(t,t^{*}\right)\right)dt^{*}. \end{array}$$ (15)

It is a boundary condition for Eq (1).

The spike duration is taken into account by introducing the time interval 0 < t* < Δ*t*_*AP*_ during which the voltage and the gating variables are fixed to their reset values. It defines the boundary conditions for Eqs (2–14) at t* = Δ*t*_*AP*_ which are as follows:

$$\begin{array}{c} U^{E}\left(t,\Delta t_{AP}\right)=V_{reset}, \end{array}$$ (16)

$$\begin{array}{c} U_{d}^{E}\left(t,\Delta t_{AP}\right)=V_{rest}; \end{array}$$ (17)

$$\begin{array}{c} \;I_{DR}:\;x\left(t,\Delta t_{AP}\right)=0.262,\;y\left(t,\Delta t_{AP}\right)=0.473; \end{array}$$ (18)

$$\begin{array}{c} \;I_{A}:\;x\left(t,\Delta t_{AP}\right)=0.743,\;y\left(t,\Delta t_{AP}\right)=0.691. \end{array}$$ (19)

The reset values for the fast gating variables in Eqs (18 and 19) were obtained with the basic single neuron model. With a rather arbitrary input providing a spike, these values were measured at the moment of a voltage maximum at the spike. The reset level for each slow conductance in the CBRD model was calculated as a sum of its value at the peak of spike-release distribution in the t*-space and an increment at spike:

$$\begin{array}{c} \;I_{M}:\;x\left(t,\Delta t_{AP}\right)=x\left(t,{t^{*}}^{p}\right)+0.175\;\left(1-x\left(t,{t^{*}}^{p}\right)\right); \end{array}$$ (20)

$$\begin{array}{c} \;I_{AHP}:\;w\left(t,\Delta t_{AP}\right)=w\left(t,{t^{*}}^{p}\right)+0.018\;\left(1-w\left(t,{t^{*}}^{p}\right)\right); \end{array}$$ (21)

where t*^p^ is such that

$$\begin{array}{c} \rho \left(t,{t^{*}}^{p}\right)\;H\left(t,{t^{*}}^{p}\right)=\max_{\;0<\;t^{*}<+\infty }\;\rho \left(t,t^{*}\right)\;H\left(t,t^{*}\right). \end{array}$$

The increment values for the slow gating variables in Eqs (20 and 21) were also measured at a single spike of the single neuron model.

#### Parameters

$$\begin{array}{ccc} V_{K} & = & -70\;\text{mV},\;\;V_{M}=-80\;\text{mV},\\ {\bar{g}}_{DR} & = & 0.76\;\mu \text{S}/cm^{2},\;\;{\bar{g}}_{A}=4.36\;\mu \text{S}/cm^{2},\\ {\bar{g}}_{M} & = & 0.4\;\mu \text{S}/cm^{2},\;\;{\bar{g}}_{AHP}=0.3\;\mu \text{S}/cm^{2},\\ \tau_{m}^{0} & = & C/g_{tot}^{0}=14.4\;\text{ms},\;\;\left(g_{L}=0.034\;\mu \text{S}/cm^{2}\right),\\ V_{rest} & = & -65\;\text{mV},\;\;\;V_{th}\left(t^{*}\right)=(-40+50\exp\left(-t^{*}/10\;\text{ms}\right)\;\text{mV},\\ V_{reset} & = & -40\;\text{mV},\;\;\;\Delta t_{AP}=1.5\;\text{ms},\\ \gamma & = & 2.85,\;\;\;C=0.7\;\mu {\text{F/cm}}^{2},\;\;\;\sigma_{V}=2\left(1+g_{syn}/g_{tot}^{0}\right)\;\text{mV},\\ S & = & 10^{-4}\;{\text{cm}}^{2} \end{array}$$

Here $g_{tot}^{0}$ is the total somatic conductance at rest, and *g*_*syn*_ is the total synaptic conductance; *S* is the membrane area. The dependency of *V*_*th*_(t*) is taken from a full single neuron model [27], allowing to take into account the effect of sodium channel inactivation on the threshold dynamics [35]. *σ*_*V*_ is the noise amplitude meaning the dispersion of individual neuron’s voltage fluctuations in a stationary state. Its scaling with *g*_*syn*_ approximately reflects the fact of the synaptic noise increase with the increase of mean synaptic drive [36].

The equations for the input synaptic conductances *g*_*GABA*,*E*_(*t*), *g*_*AMPA*,*E*_(*t*) and *g*_*NMDA*,*E*_(*t*) are given in Section 2.5, as well as the values of the reversal potentials.

When calculating the dynamics of a neural population, the integration of Eqs (2–14) determines the evolution of the distribution of voltage *U*^*E*^ across t*. Then, the effect of crossing the threshold and the diffusion due to noise are taken into account by *H*-function, Eq (4), substituted into the equation for neuronal density (1). The integral (15) results in the output firing rate *ν*^*E*^(*t*).

Two compartment model of principal neurons allows calculating the local field potential (LFP) originating from the dipole-like configuration of membrane currents. Such formula for LFP signal has been derived in [32] and is used in the present work.

### 2.3 Modeling: CBRD-approach for a population of interneurons

Here we describe the CBRD-model for an inhibitory population labeled as “*I*” and omit this index throughout this section wherever possible. Taking into account the majority of interneuron subtypes, we use one of the simplest models for interneurons, which originates from the single-compartment model for fast spiking interneurons [37]. It was parameterized by t* in our previous work (Chizhov 2013), similar to the model for the excitatory population from Section 2.2:

$$\begin{array}{ccc} \frac{\partial \rho^{I\;}}{\partial t} & + & \frac{\partial \rho^{I\;}}{\partial t^{*}}=-\rho^{I\;}H\left(U^{I\;},g_{tot}^{I\;}\right), \end{array}$$ (22)

$$C\;(\frac{\partial U^{I\;}}{\partial t}+\frac{\partial U^{I\;}}{\partial t^{*}})=-g_{L}\left(U^{I\;}-V_{rest}\right)-I_{K}+g_{AMPA,I\;}\left(t\right)\left(V_{AMPA}-U^{I\;}\right)+g_{GABA,I\;}\left(t\right)\left(V_{GABA}-U^{I\;}\right)+g_{G}J\left(U_{mean}^{I\;}-U^{I\;}\right)$$ (23)

#### Ionic channels

The transmembrane current includes the voltage-dependent potassium current *I*_*K*_ [37], approximated as follows:

$$\begin{array}{ccc} I_{K}\left(U^{I}\;,t,t^{*}\right) & = & {\bar{g}}_{K}\;n^{4}\left(t\right)\;\left(U^{I}\;\left(t\right)-V_{K}\right), \end{array}$$ (24)

$$\begin{array}{c} \frac{\partial n}{\partial t}+\frac{\partial n}{\partial t^{*}}=\frac{n_{\infty }\left(U^{I}\;\right)-n}{\tau_{n}}, \end{array}$$ (25)

$$\begin{array}{ccc} & & \tau_{n}=\left(0.5+2/\left(1+\exp\left(0.045\;\left(U^{I}\;-50\right)\right)\right)\right)\;\text{ms},\\ & & n_{\infty }=1/\left(1+\exp\left(-0.045\;\left(U^{I}\;+10\right)\right)\right). \end{array}$$

The gap-junctions between interneurons were taken into account in Eq (23) with the mean voltage

$$\begin{array}{c} U_{mean}^{I\;}\left(t\right)=\int_{0}^{\infty }\;U^{I\;}\left(t,t^{*}\right)\;\rho \left(t,t^{*}\right)\;dt^{*}. \end{array}$$ (26)

#### Boundary conditions

$$\begin{array}{c} \nu^{I}\;\left(t\right)\equiv \rho^{I}\;\left(t,0\right)=\int_{+0}^{\infty }\;\rho^{I}\;\left(t,t^{*}\right)H\left(U^{I}\;\left(t,t^{*}\right)\right)dt^{*}, \end{array}$$ (27)

$$\begin{array}{c} U^{I}\;\left(t,\Delta t_{AP}\right)=V_{reset},\;\;\;n\left(t,\Delta t_{AP}\right)=0.45 \end{array}$$ (28)

#### Parameters

$$\begin{array}{ccc} V_{K} & = & -80\;\text{mV},\;\;\;{\bar{g}}_{K}=40\;{\text{mS/cm}}^{2},\\ g_{L} & = & 0.1\;\mu \text{S}/cm^{2},\;\;\;\left(\tau_{m}^{0}=C/g_{tot}^{0}=9.7\;\text{ms}\right),\\ V_{rest} & = & -60\;\text{mV},\;\;\;V_{reset}=-40\;\text{mV},\\ \;\;\;V_{th}\left(t^{*}\right) & = & (-50+20\exp\left(-t^{*}/10\;\text{ms}\right)\;\text{mV},\\ \;\;\;\Delta t_{AP} & = & 1.4\text{ms},\;\;\;\gamma =2.85,\;\;\;C=1\;\mu {\text{F/cm}}^{2},\\ \sigma_{V} & = & 3\left(1+g_{syn}/g_{tot}^{0}\right)\;\text{mV},\;\;\;S=10^{-4}\;{\text{cm}}^{2} \end{array}$$

Stochastic input was modeled as Ornstein-Uhlenbeck process with the time correlation 4 ms and the dispersion 20 pA.

### 2.4 Modeling: Lognormal distribution of synaptic weights within each population

To introduce realistic, lognormal distribution of synaptic weights within a population *j* (*E* or *I*), the CBRD-approach has been generalized in [31]. In this case, instead of the equal total synaptic current, neurons receive lognormally distributed current. For the current scaled by its mean across the distribution, *x*, the distribution is

$$\begin{array}{c} \psi \left(x\right)=\frac{\exp(-{\left(\ln x\right)}^{2}/\left(2\;\sigma_{LN}^{2}\right))}{\sqrt{2\pi }\;\sigma_{LN}\;x} \end{array}$$ (29)

The membrane potential of neurons parameterized with *x*, $U_{x}^{j}$, can be found as

$$\begin{array}{c} U_{x}^{j}\left(t,t^{*}\right)=\left(U^{j}\left(t,t^{*}\right)-U_{free}^{j}\left(t^{*}\right)\right)\;x+U_{free}^{j}\left(t^{*}\right), \end{array}$$ (30)

where $U_{free}^{j}\left(t^{*}\right)$ is the unperturbed potential defined for zero synaptic input.

The density of neurons parameterized by *x* and distributed in the phase space t* is denoted as $\rho_{x}^{j}\left(t,t^{*}\right)$. Calculation of $\rho_{x}^{j}\left(t,t^{*}\right)$ requires solving of a continuous set of Eq (1) (or Eq (22)) for $\rho_{x}^{j}$ instead of *ρ*^*j*^ with $H(U_{x}^{j},dU_{x}^{j}/dt)$. The output firing rate is defined as

$$\begin{array}{c} \nu^{j}\left(t\right)=\int_{0}^{\infty }\;\rho_{x}^{j}\left(t,0\right)\;\psi \left(x\right)\;dx \end{array}$$ (31)

In numerical simulations, we set the parameter of the lognormal distribution *σ*_*LN*_ = 0.5 and discretized the *x*-space by 10 intervals.

### 2.5 Modeling: Connections

The synaptic conductances are described following [28] with introduced synaptic plasticity factors $x_{glu}^{D}\left(t\right)$ and $x_{GABA}^{D}\left(t\right)$, i.e. as follows

$$\begin{array}{c} g_{AMPA,j}\left(t\right)={\bar{g}}_{AMPA,j}\;m_{AMPA,j}\left(t\right)\;x_{glu}^{D}\left(t\right), \end{array}$$

$$\begin{array}{c} g_{NMDA,j}\left(t\right)={\bar{g}}_{NMDA,j}\;f_{NMDA}\left(U^{j}\left(t\right)\right)\;m_{NMDA,j}\left(t\right)\;x_{glu}^{D}\left(t\right), \end{array}$$ (32)

$$\begin{array}{ccc} f_{NMDA}(V) & = & 1/(1+Mg/3.57\;\text{exp}(-0.062V)),\\ & & \text{for}\;\;j=E\;\text{and}\;I, \end{array}$$ (33)

$$\begin{array}{ccc} g_{GABA,j}\left(t\right) & = & {\bar{g}}_{GABA,j}\;m_{GABA,j}\left(t\right)x_{GABA}^{D}\left(t\right),\\ \;\; & & \text{for}\;\;j=E\;\text{and}\;I\; \end{array}$$ (34)

*Mg* is the magnesium (Mg^2+^) concentration in mM; *m*_*s*,*j*_(*t*) is the non-dimensional synaptic conductance which is approximated by the second order ordinary differential equation:

$$\left(\tau_{r}^{s,j}\tau_{d}^{s,j}\frac{d^{2}}{dt^{2}}+(\tau_{r}^{s,j}+\tau_{d}^{s,j})\frac{d}{dt}+1\right)\;m_{s,j}(t)=\tau^{s,j}\left(1-m_{s,j}(t)\right)\varphi_{i}(t),$$ (35)

$$\begin{array}{ccc} \tau^{s,j} & = & (\tau_{r}^{s,j}-\tau_{d}^{s,j})/({\left(\tau_{d}^{s,j}/\tau_{r}^{s,j}\right)}^{\tau_{d}^{s,j}/(\tau_{r}^{s,j}-\tau_{d}^{s,j})}-{\left(\tau_{d}^{s,j}/\tau_{r}^{s,j}\right)}^{\tau_{r}^{s,j}/(\tau_{r}^{s,j}-\tau_{d}^{s,j})}),\\ & & \;\;\text{if}\;\;\tau_{r}^{s,j}\neq \tau_{d}^{s,j},\\ & & \tau_{r}^{s,j}e,\;\;\text{otherwise}. \end{array}$$ (36)

Here ϕ_*i*_ is the presynaptic firing rate. In neglect of spatial propagation and temporal delays the presynaptic firing rate is equivalent to the somatic firing rate, i.e. *φ*_*i*_ ≡ *ν*_*i*_. The index *s* is the synapse type, *s* = *AMPA*, *GABA* or *NMDA*; the index *i* = *E* for *s* = *AMPA* or *NMDA* and *i* = *I* for *s* = *GABA*; *w*_*glu*_ and *w*_*GABA*_ are the synaptic weights that change because of short-term plasticity; ${\bar{g}}_{s,j}$ is the maximum conductance, $\tau_{r}^{s,j}$ and $\tau_{d}^{s,j}$ are the rise and decay time constants. We imply that the synaptic time constants are estimated from the somatic responses to the stimulation of a presynaptic neuronal population, thus these time constants characterize not only synaptic channel kinetics but the dendritic and axonal propagation delays as well. The time scale τ^*s*,*j*^ is chosen in the form of Eq (36) in order to provide independence of the maximum of *g*_*s*,*j*_(*t*) on $\tau_{r}^{s,j}$ and $\tau_{d}^{s,j}$, when *g*_*s*,*j*_(*t*) is evoked by a short pulse of *φ*_*j*_(*t*).

The parameter values were as follows: ${\bar{g}}_{AMPA,E}={\bar{g}}_{NMDA,E}=0.6\;\mu \text{S}/cm^{2}$, ${\bar{g}}_{GABA,E}={\bar{g}}_{GABA,I\;}=0.5\;\mu \text{S}/cm^{2}$, ${\bar{g}}_{AMPA,I\;}={\bar{g}}_{NMDA,I\;}=0.2\;\mu \text{S}/cm^{2}$, *V*_*AMPA*_ = *V*_*NMDA*_ = 0, *Mg* = 0.25 mM, $\tau_{r}^{AMPA,E}=\tau_{r}^{AMPA,I\;}=1.7\;\text{ms}$, $\tau_{d}^{AMPA,E}=\tau_{d}^{AMPA,I\;}=8.3\;\text{ms}$, $\tau_{r}^{NMDA,E}=\tau_{r}^{NMDA,I\;}=6.7\;\text{ms}$, $\tau_{d}^{NMDA,E}=\tau_{d}^{NMDA,I\;}=100\;\text{ms}$, $\tau_{r}^{GABA,E}=\tau_{r}^{GABA,I\;}=0.5\;\text{ms}$, $\tau_{d}^{GABA,E}=\tau_{d}^{GABA,I\;}=20\;\text{ms}$, *g*_*GJ*_ = 0.2 *μ*S/*cm*^2^.

### 2.6 Modeling: Synaptic plasticity

The synaptic depression was modeled with the equation from [38]

$$\begin{array}{ccc} \frac{dx_{glu}^{D}}{dt} & = & \frac{\left(1-x_{glu}^{D}\right)}{\tau_{glu}}-U_{glu}\;x_{glu}^{D}\;\varphi_{E}\left(t\right), \end{array}$$ (37)

$$\begin{array}{ccc} \frac{dx_{GABA}^{D}}{dt} & = & \frac{\left(1-x_{GABA}^{D}\right)}{\tau_{GABA}}-U_{GABA}\;x_{GABA}^{D}\;\varphi_{I\;}\left(t\right), \end{array}$$ (38)

with *τ*_*glu*_ = *τ*_*GABA*_ = 2000 ms and *U*_*glu*_ = *U*_*GABA*_ = 0.04.

### 2.7 Modeling: Numerical approach

The transport equations with the independent variables *t* and t* (Eqs (1–3, 6, 7, 9, 10, 12, 14) for the excitatory population and Eqs (22, 23, 25) for the inhibitory population) can be solved with numerical scheme constructed in the framework of either the Eulerian [27] or Lagrangian description. In the latter case, the semi-infinite t*-space is bounded by the interval [0, *B*] and discretized by *N* intervals. Each *i*-indexed interval is represented by a non-spiking probe neuron, initially located at $t_{i}^{*}$. Each probe neuron *i* represents a certain fraction of a population, quantified by $\rho (t,t_{i}^{*})$. The states of the probe neurons (the voltage and gating variables) and the neuronal density attributed to probe neurons evolve according to the main transport equations with the total derivative in time in the left-hand part. Their t*-coordinates increase accordingly to time *t* up to *B*. If a probe neuron reaches t* = *B*, its t*-coordinate is renewed to 0, their potential and gating variables are replaced or incremented in accordance with the boundary conditions. The neuronal density at t* = 0 is equal to the flux *ρH* accumulated during the time *B*/*N*. In the present study, this approach has been applied with the parameters *B*^*E*^ = 100 ms, *N*^*E*^ = 100, *B*^*I*^ = 40 ms, *N*^*I*^ = 50.

## 3 Results

### 3.1 Experimental observations of IIDs

**Fig 1:**
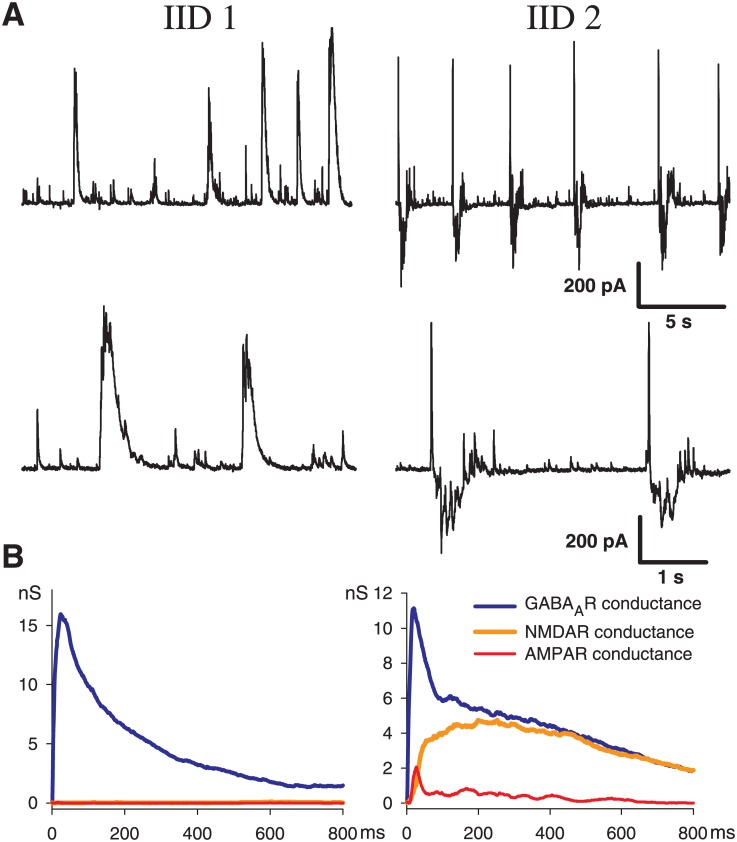
Experiment. Interictal discharges in combined entorhinal cortex-hippocampal slices. A: IID1 (left) and IID2 (right) recorded in voltage-clamp at (*V*_*hold*_ = −27 mV). B: synaptic conductances underlying the discharges, estimated with the method from [14].

As reported previously [14], after perfusion of the brain slices containing the entorhinal cortex and hippocampus with eS, two types of interictal discharges were observed. Representative examples of IID1 and IID2, recorded at a holding voltage of -27 mV in entorhinal pyramidal neurons, are shown in Fig 1. At this membrane potential, the GABAergic current is outward, whereas the glutamatergic AMPAR- and NMDAR-mediated currents are inward. IID1s are composed of purely outward (positive) currents; IID2s have both positive and negative components. The frequency of IID1s was 0.22 ± 0.04 Hz (n = 29), which does not differ from that of IID2s (0.24 ± 0.02 Hz (n = 22)). However, IID2s occurred more regularly than IID1s, as suggested by the coefficients of variation for frequency (CV = 0.98 and 0.39 for IID1s and IID2s, respectively). It was revealed that during IID1 activity, pyramidal cells receive only GABAergic input (Fig 1, bottom left). During IID2s activity the synaptic inputs were complex, and the GABA component was followed by AMPA and NMDA components (Fig 1, bottom, right). The NMDA component was highly pronounced in the later phase of IID2s. Within each of the recorded traces, the amplitudes and durations of IID1s as well as the interburst interval were more variable for IID1s than for IID2s.

**Fig 2:**
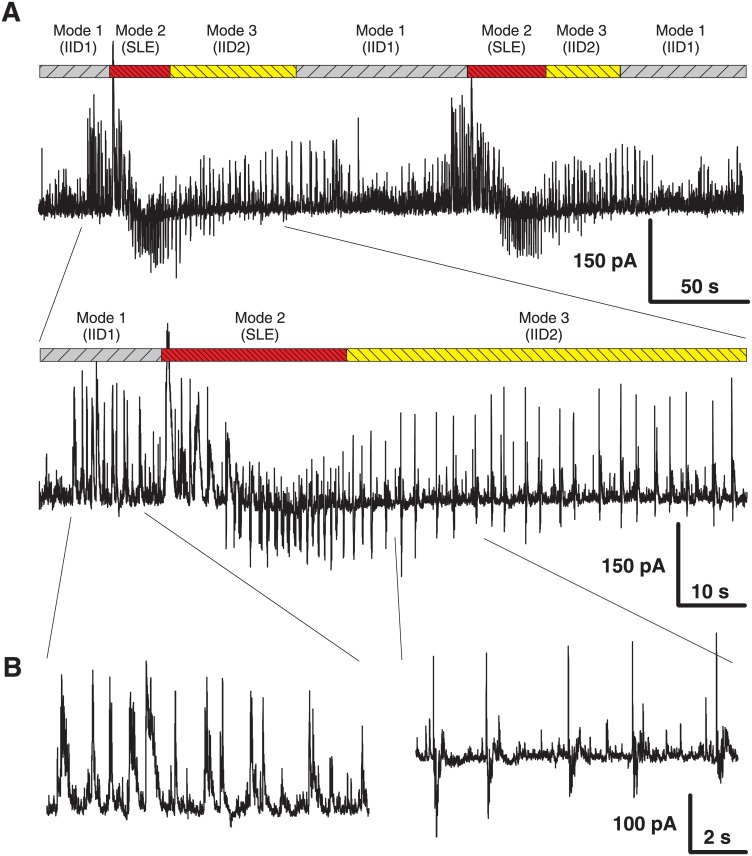
Experiment. Ictal and interictal discharges in voltage-clamp (*V*_*hold*_ = −27 mV) in isolated entorhinal cortex slice. A: Transitions between 3 modes of synchronized synaptic activity in the isolated cortex: IID1, seizure-like events (SLEs) and IID1. B: Expanded traces from A with the representative current patterns.

Because the generation of epileptiform discharges in the entorhinal cortex might be affected by the influence of hippocampal neurons, the entorhinal cortex area was isolated. Areas of the subiculum and hippocampus were cut off. Similar to the observations in the full slices [14], three modes of synchronized network activity have been observed (Fig 2A). The first mode contained only IID1. In most of the slices, IID1 were replaced with the second mode with ictal discharges (or seizure-like events). The third mode was characterized by IID2 that emerged more regularly than IID1. The transitions between modes of the synchronized synaptic activities were observed in the majority of slices; however, in some slices, the first mode of activity was the only one, and in other slices, the activity skipped ictal discharges and proceeded directly to the third mode.

It was found that the properties of interictal discharges in the entorhinal cortex (Fig 2B) were similar to those in the combined slices (Fig 1A), and it was concluded that the entorhinal cortex alone is sufficient to generate and maintain epileptiform activity. Therefore, the effects of spatial propagation or interactions between different areas were not considered in the modeling study.

### 3.2 Mechanisms of IIDs and validation of model assumptions

**Fig 3:**
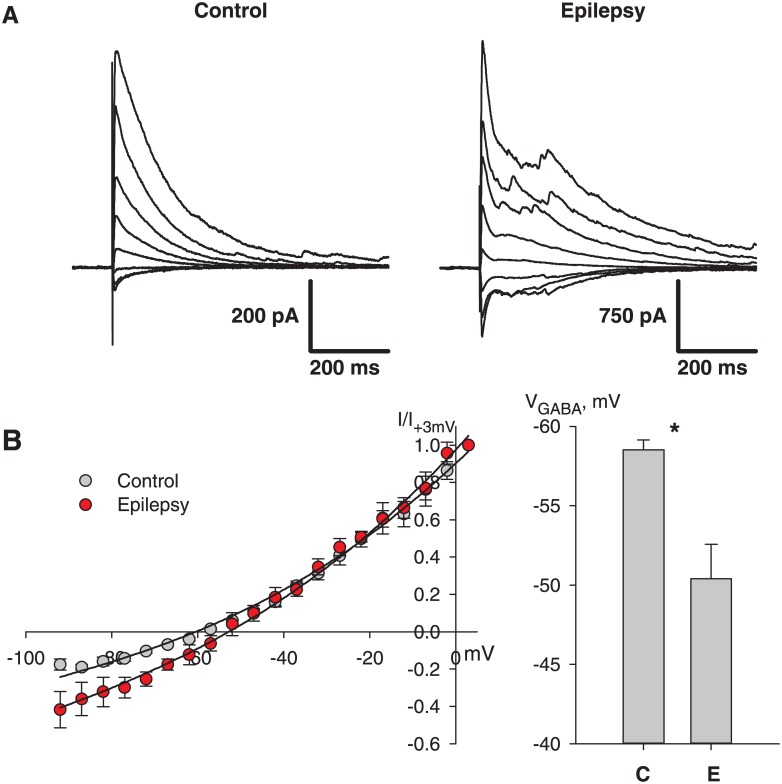
Experiment reveals a shift of GABA reversal potential. A: IPSCs were recorded in control and epileptic solution with pharmacological blockade of glutamatergic synaptic currents at different holding potentials. B: left, the I-V relationships were plotted for the peak currents shown in A. Right, the statistical estimations of *V*_*GABA*_ reveal reliable shift from -58 in control up to -50 mV in pro-epileptic solution. Experimental data points are fitted with the Goldman-Hodgkin-Katz equation as in [14].

It is hypothesized that the mechanism of IIDs includes interactions between excitatory and inhibitory neurons by chemical GABA- and glutamatergic synapses as well as electrical synapses between inhibitory neurons. It is also suggested that IID generation is caused by changes in the extracellular potassium and intracellular chloride concentrations. Indeed, the eS has a higher extracellular potassium concentration. To test the assumption that the application of eS changes the intracellular chloride concentration and shifts *V*_*GABA*_ in neurons, we recorded the evoked IPSCs in the Ringer’s solution 5 cells from 5 slices) and in eS (6 cells from 6 slices) (Fig 3A). The peak values of the currents versus the holding voltage were plotted for each neuron, as shown in the left plot in Fig 3B. The reversal potential *V*_*GABA*_ was evaluated by fitting the IV-relationship with Goldman-Hodgkin-Katz flux equation. The obtained values were statistically compared with the t-test, *V*_*GABA*_ were significantly different in the two cases (*p* < 0.05). Mean *V*_*GABA*_ changed from -58 mV to -50 mV in eS (Fig 3B). Because there is an exchange of chloride ions with the pipette solution in whole-cell configuration, the shift in *V*_*GABA*_ might be even larger in other cells of the slice [39]. Thus, the experimental data supports the assumption regarding the shift of *V*_*GABA*_ in the proepileptic solution.

Concentrations of chloride and potassium ions were assumed to be constants within each type of IIDs because the variation in the ionic concentrations is not significant during IID activity, whereas the interburst periods are long enough to provide ionic relaxation between IIDs (see [12], [40] for potassium, and [41] for chloride).

Fu and van den Pol (2007) revealed strongly depolarized GABAA reversal potential (*V*_*GABA*_ = -35 mV) in a subset of hippocampal interneurons. Therefore, GABAergic excitation within the subset of neurons led to pure GABA-mediated spontaneous discharges [42]. Viitanen et al. [43] determined that after high-frequency stimulation, the shift of *V*_*GABA*_ reached 30 mV in CA1 hippocampal neurons. Based on these and other observations [44], [13], [45], [18], [46], [47], for the simulations, the depolarized values for *V*_*GABA*_ during IIDs were assumed. For IID1s *V*_*GABA*_ was set as equal to -50 mV for both types of neurons. Moreover, because the prolonged activity of interneurons may change intracellular chloride concentrations in neurons, shifting *V*_*GABA*_ to a more depolarized value, *V*_*GABA*_ for IID2s was assumed to be even more depolarized at up to -45 mV. Thus, it was assumed that a change in *V*_*GABA*_ is the most significant factor that determines the preferred type of IIDs.

The experimental data revealed that both types of IIDs triggered by the spontaneous firing of interneurons and the synchronization of interneurons precedes the synchronization of pyramidal neurons. These results suggest that the depolarizing effect of GABA is stronger in interneurons compared with pyramidal cells. Several factors might underlie this discrepancy between neuron populations, including the difference in GABAergic synaptic conductance, the spiking threshold, input resistance, *V*_*GABA*_, or the resting potential. Two factors are suggested to be the most significant. First, some populations of interneurons may exhibit a low threshold of firing, such as spontaneously (intrinsically) spiking interneurons, which are presumably somatostatin-expressing GABAergic neurons [24]. Second, in at least some subsets of interneurons, the chloride concentration is higher than in pyramidal cells [42]. To take into account a cumulative effect of these factors in the mathematical model, the firing threshold for interneurons was set lower than for excitatory pyramidal cells (-50 mV versus -40 mV, correspondingly). The remaining parameters, which determine excitability, were assumed to be similar for both neuronal populations. These parameters include the GABAergic synaptic conductance, the input conductance, VGABA, and the resting membrane potential.

### 3.3 IID1s as a recurrent GABAergic excitation of interneurons

**Fig 4:**
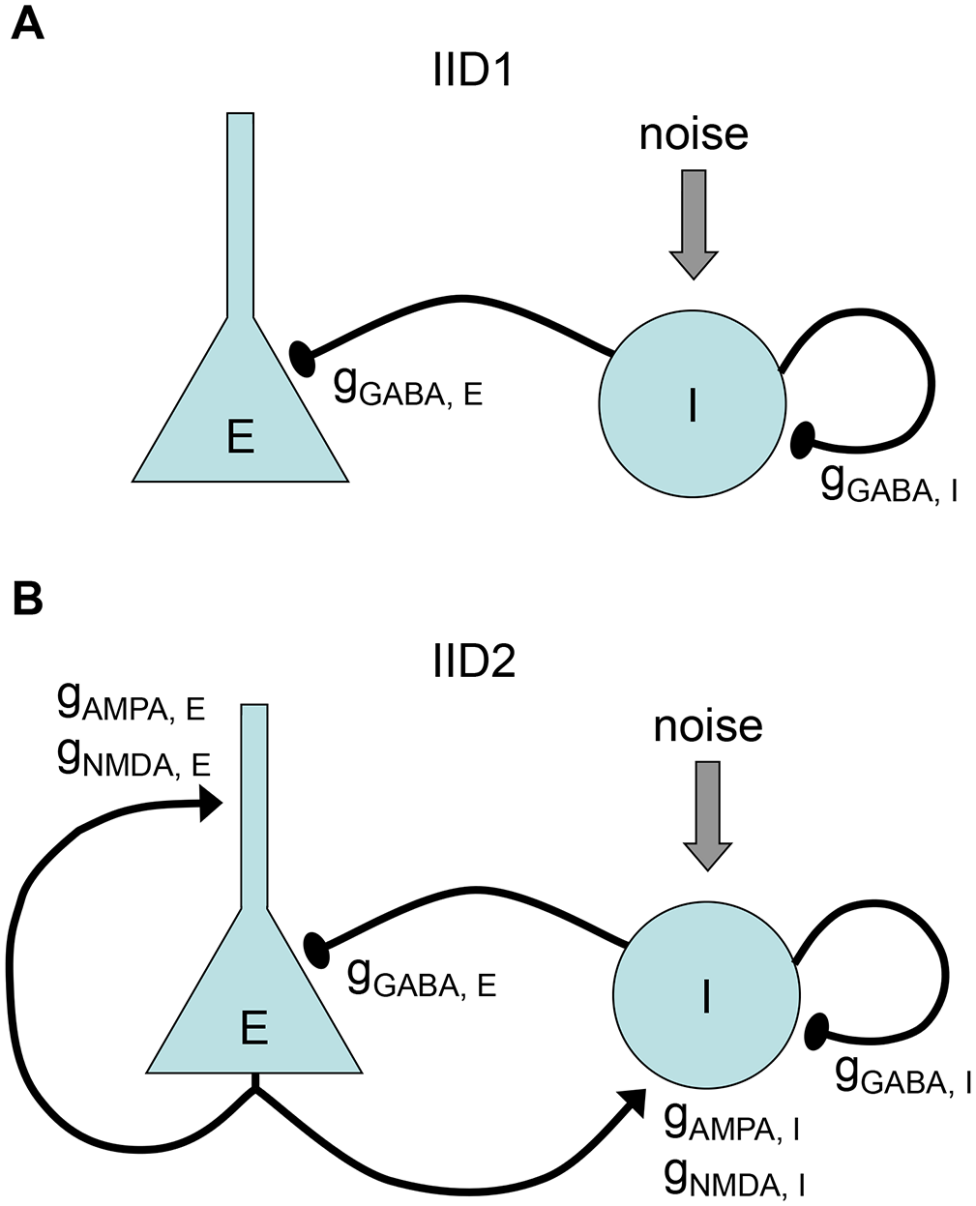
Schematic of connections between populations of excitatory neurons (E) and interneurons (I) involved in the generation of IID1 and IID2. The excitatory neurons have two, somatic and dendritic compartments, which receive GABAergic and glutamatergic inputs. The single-compartment interneurons receive glutamatergic input, noisy excitatory input-current and recurrent GABAergic drive. A, During IID1s the excitatory neurons do not fire. Interneurons receive recurrent excitation through GABAergic synapses. This case is modeled by setting *V*_*GABA*_ = −50 mV. B, During IID2s the excitatory neurons are triggered by spontaneous activity of interneurons by means of depolarizing GABAergic synapses. This case is modeled by setting *V*_*GABA*_ = −45 mV. Note that the schematic in A includes only the connections that are activated during IID1, whereas during IID2 all those connections that are included in the model are activated, as plotted in B.

Based on the experimental observations, including the previously reported data [14], the mechanism of IID1 generation implies spontaneous activity of interneurons that result in synchronous, pure GABAergic synaptic events in principle neurons (Fig 1). It was presumed that the synchronization of interneurons occurs due to the excitatory effect of GABA in the recurrent interneuron network. This effect occurs after chloride accumulation in the cytoplasm, which in turn is a consequence of the reduced or reversed action of K-Cl-transporters after the application of high-potassium eS. The schematics of the model network with the main synaptic connections involved in the generation of IID1 is illustrated in Fig 4A. Other synaptic connections (compare Fig 4A with Fig 4B) do not participate.

**Fig 5:**
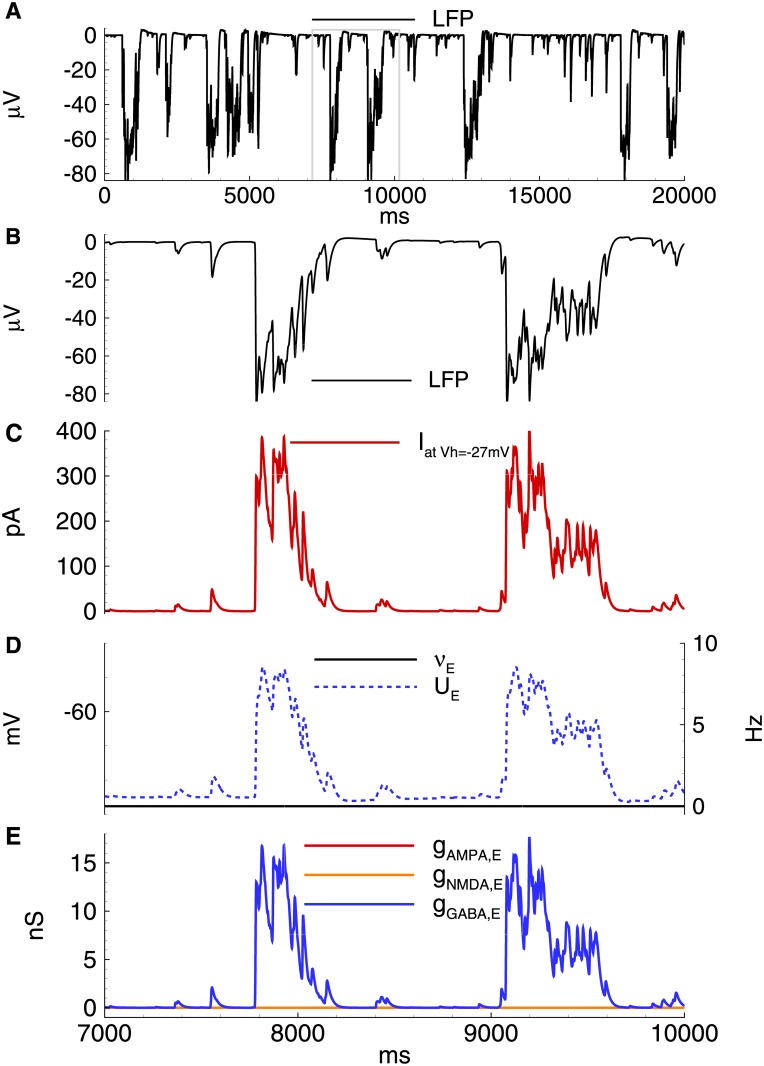
Model. IID1s. A: Local field potential (LFP) at the interval of 32s after the beginning of spontaneous current injection (20 pA amplitude, 4 ms correlation time). B-E: characteristics of the discharges on 2.4 s interval: B: LFP. C: the membrane current at *V*_*hold*_ = −27mV. D: the firing rate and the mean membrane potential. E: the synaptic conductances. *V*_*GABA*_ = −50 mV.

**Fig 6:**
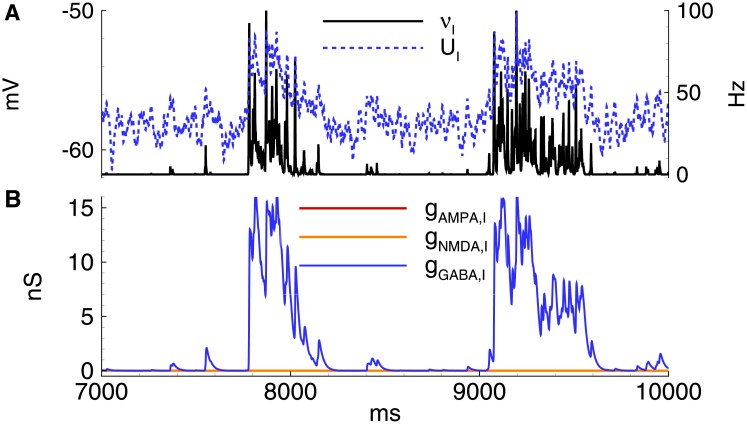
Model. The activity of interneurons during IID1s. A: the firing rate and the mean membrane potential. B: the synaptic conductances. The simulation corresponds to that in Fig 5. *V*_*GABA*_ = −50 mV.

According to the model, *V*_*GABA*_ determines the development of IID1. Its value should be close to the threshold voltage of the spike generation for the interneurons. For IID1s, *V*_*GABA*_ was set −50 mV, which is equal to the minimum spiking threshold value in interneurons (see Section 2.3). During the simulation (Figs 5 and 6), the membrane voltage fluctuated due to introduced noise, which reflects spontaneous synaptic activity (Fig 6A). Voltage fluctuations rarely reach the spiking threshold. Therefore, interneurons may generate spontaneous spikes, which evoke depolarizing GABAergic postsynaptic potentials (GABA-PSP) (Figs 5 and 6). If some interneurons in the population fire, then the excitation provides positive feedback within the short time of the decay of GABA-PSP and results in the synchronous firing of the entire interneuron population. On the contrary, if the GABA-PSPs do not coincide, then the membrane potential remains subthreshold and decays further.

As a result, two types of spontaneous discharges (Fig 5A) were observed, including small events corresponding to mono- or double-synaptic GABA-PSPs and large events determined by the synchronized activity of the interneurons. All discharges were initiated by the GABAergic interneurons and were observed as outward currents at -27 mV (Fig 5C). Only GABAergic synaptic responses were observed in both the pyramidal cells and interneurons (Figs 5E and 6B). The GABA-mediated currents depolarized both types of neurons (Figs 5D and 6A); however, only interneurons generated action potentials (Fig 5A).

Whereas in our experiments the synchronous character of the activity was revealed by double patch-clamp recordings [14], it is conventionally verified using field recordings. That is why we model the local field potentials (LFP). The simulated LFP during IIDs (Fig 5A and 5B) are similar to the experimental recordings [13], [12].

**Fig 7:**
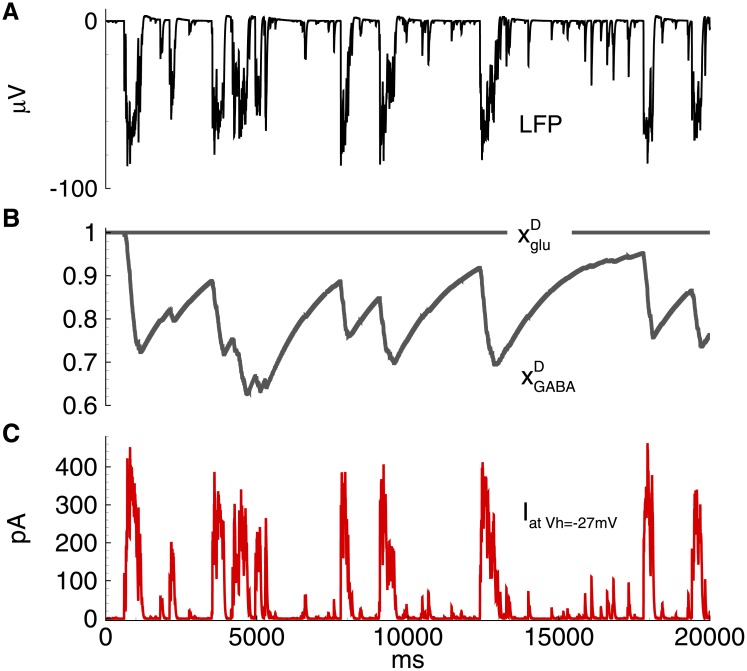
Model. IID1s. Short-term synaptic depression. A: field potential. B: short-term synaptic depression variables $x_{glu}^{D}$ and $x_{GABA}^{D}$. C: the membrane current at *V*_*h*_ = −27mV in a representative excitatory neuron. The simulation corresponds to that in Fig 5.

The model predicts that the frequency and duration of IID1s are determined by the parameters of short-term synaptic depression, *τ*_*GABA*_, and *U*_*GABA*_. The synaptic resource $x_{GABA}^{D}$ rapidly decreases beginning from the onset of each IID1 as much as determined by *U*_*GABA*_ and then recovers on the interburst intervals with the characteristic time *τ*_*GABA*_ (Fig 7B).

In summary, it was found that the proposed model provides the following experimental observations (Fig 1):

- the stochastic character of IID1s (Fig 1, top, left from the experiment and Fig 5A from the simulation)
- the synchronous character of the discharges (correlation of the current in a single neuron [Fig 5C] with the population firing rate [Fig 5B]);
- interneurons are necessary and sufficient for IID1 generation and its maintenance (Fig 1, bottom, left and Fig 5E);
- the amplitude and duration of the average IID1.

**Fig 8:**
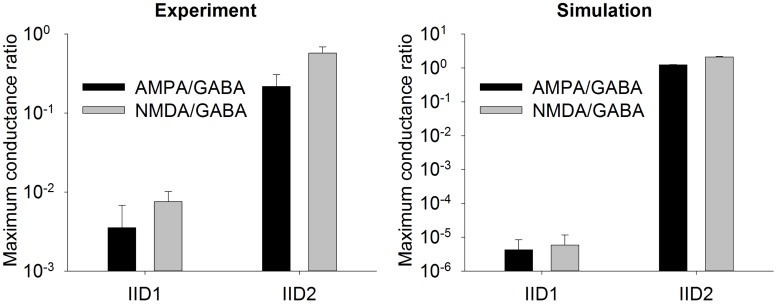
Experiment and model. Mean ratios of maximum synaptic conductances during IID1 and IID2. The statistics is done for averaged IID1 from 5 cells and averaged IID2 from 10 cells in experiment, and 107 IID1 and 32 IID2 in simulation.

As described above, the most distinguished feature of IID1 is a small ratio of excitatory to GABAergic synaptic conductances (Fig 1B). Statistically treated data on the ratios of maximum conductances during IID1 events (Fig 8) show the consistency between the model and experiments. Because of such small contribution of the excitatory connections, only GABA-ergic connections are included in the schematic of the mechanism of IID1, depicted in Fig 4A.

### 3.4 IID2s as a recurrent excitation triggered by the stochastic activation of depolarizing inhibition

According to the experimental observations, the mechanism of IID2 initiation is similar to that of IID1, but the maintenance of these discharges is different. It was assumed that for IID2s, the synchronous activity of interneurons induces a stronger depolarization of neighboring neurons because *V*_*GABA*_ is more depolarized. *V*_*GABA*_ is close to the action potential threshold of pyramidal cells, and therefore interneurons may evoke the firing of pyramidal cells. The depolarization is further amplified by the recurrent excitatory connections, thus resulting in strong glutamatergic excitation. Hence, the mechanism of IID2 includes interacting populations of the excitatory neurons and the interneurons driven by a noisy input, as shown in the schematic illustration in Fig 4B.

Several experimental studies have provided evidence that supports the suggestion that a shift of *V*_*GABA*_ determines the type of IID. First, IID2s were usually detected after ictal discharges, whereas IID1s were detected before [14]. Ictal events increase *V*_*GABA*_, which has been shown in multiple studies [48], [41].

**Fig 9:**
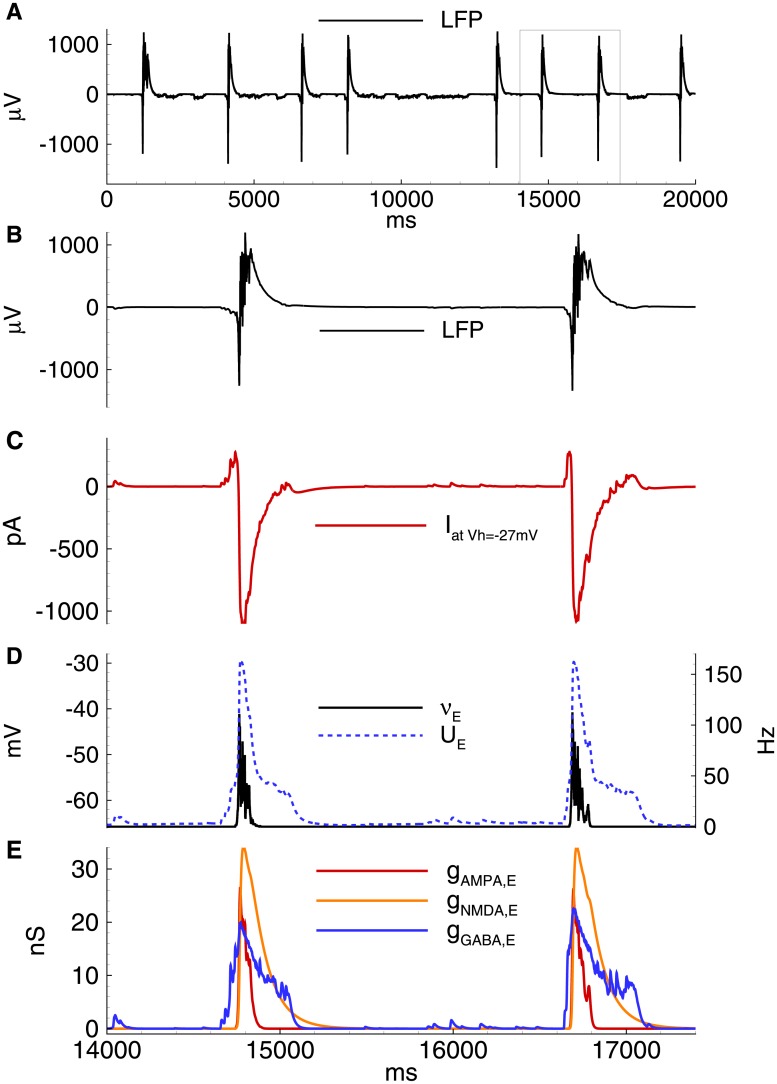
Model. IID2s. A: Local field potential (LFP) at the interval of 32s after the beginning of spontaneous current injection (20 pA amplitude, 4 ms correlation time). B-E: characteristics of the discharges on 2.4 s interval: B: LFP. C: the membrane current at *V*_*hold*_ = −27mV. D: the firing rate and the mean membrane potential. E: the synaptic conductances. *V*_*GABA*_ = −45mV.

**Fig 10:**
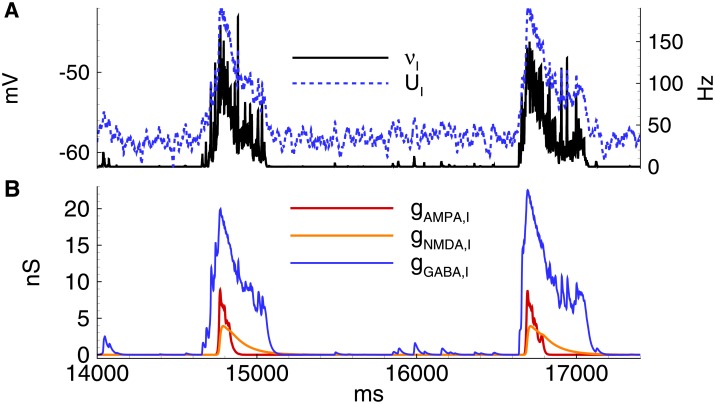
Model. IID2s. Activity of interneurons. A: the firing rate and the mean membrane potential. B: the synaptic conductances. *V*_*GABA*_ = −45 mV. The simulation corresponds to that in Fig 9.

**Fig 11:**
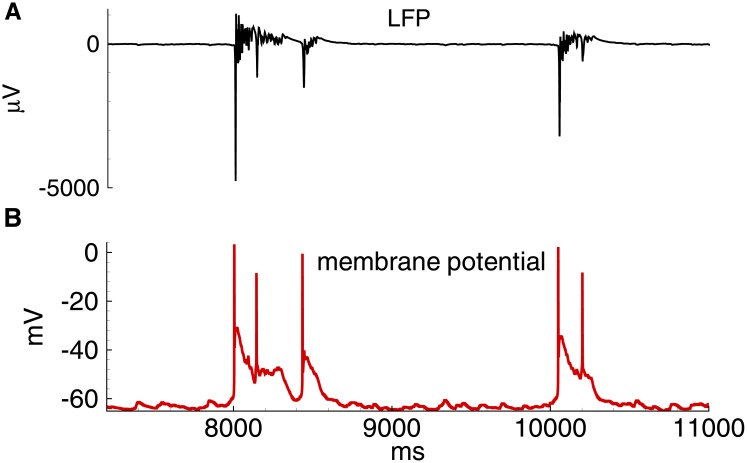
Model. Field potential and membrane voltage during IID2s. A: field potential. B: membrane voltage in a representative excitatory neuron.

For IID2 with *V*_*GABA*_ = -45 mV in the simulation of the 20 s epoch, a number of spontaneous discharges was observed (Fig 9A). All were initiated by the GABAergic interneurons (Fig 9C). These GABA inputs depolarize both pyramidal cells and interneurons (Figs 9D and 10A), and both populations become active (Figs 9D and 10A). The firing activity of excitatory cells results in the activation of AMPA and NMDA conductances. The early outward current recorded at -27 mV shifts to an inward current due to the dominant contribution of AMPA and NMDA-mediated currents. A typical excitatory neuron generates a few spikes during each IID2 (Fig 11).

The activity of interneurons during IID2s is qualitatively similar to that of the principal cells (Fig 10). It is important to note that not all spontaneous GABA-mediated events are IID2s. An IID may occur if some time passed since a previous discharge due to the refractoriness provided by short-term synaptic depression.

**Fig 12:**
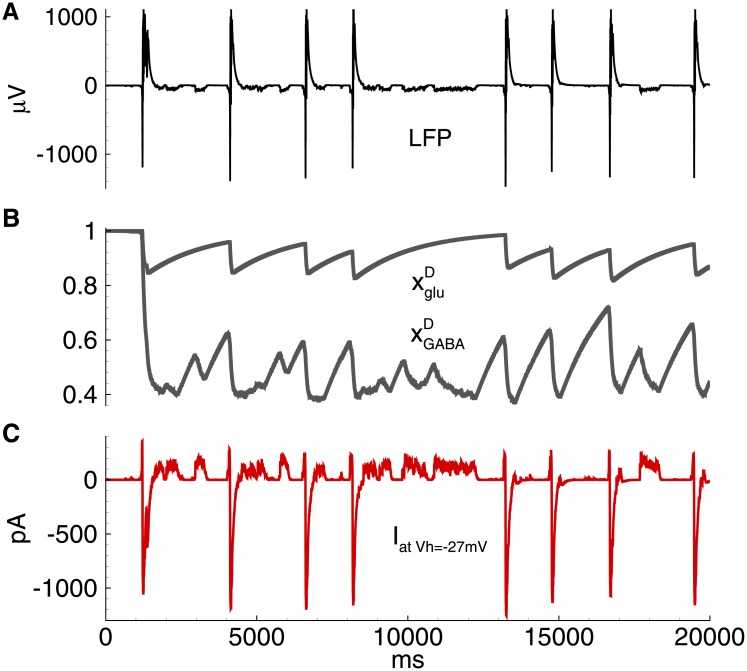
Model. IID2. Short-term synaptic depression. A: field potential. B: short-term synaptic depression variables $x_{glu}^{D}$ and $x_{GABA}^{D}$. C: the membrane current at *V*_*h*_ = −27mV in a representative excitatory neuron. The simulation corresponds to that in Fig 9.

The model predicted that the duration of IID2s and their interburst intervals are determined by the parameters of the short-term depression of GABA and glutamatergic synapses, *τ*_*glu*_, *τ*_*GABA*_, *U*_*glu*_, and *U*_*GABA*_. The synaptic resources $x_{GABA}^{D}$ and $x_{glu}^{D}$ rapidly decreases with each IID2 and then recovers on the interburst intervals with the characteristic times *τ*_*glu*_ and *τ*_*GABA*_ (Fig 12B).

In summary, the proposed model illustrated the following experimentally observed properties of IID2s:

- the stochastic character of IID2 generation with a characteristic interburst interval of about a few seconds (compare Fig 1, top, right and Fig 9C);
- the synchronous character of the discharges (correlation of the current in a single neuron [Fig 9C] with the population firing rate [Fig 9B]);
- a featured shape of the current recorded at the voltage level -27 mV with an initial outward phase and a later inward phase (Fig 1, bottom, right and Fig 9E;)
- the amplitude and the duration of an average IID2.

As described above, the most distinguished feature of IID2 is a comparable contribution of excitatory and GABAergic conductances (Fig 1). Mean ratios of maximum conductances during IID2 events, given in Fig 8, are significantly different from those for IID1 in the experiments and simulations. Similar contributions of excitatory and GABAergic conductances are taken into account in the schematic mechanism of IID2s, that is depicted in Fig 4B.

### 3.5 Effects of short-term synaptic plasticity

**Fig 13:**
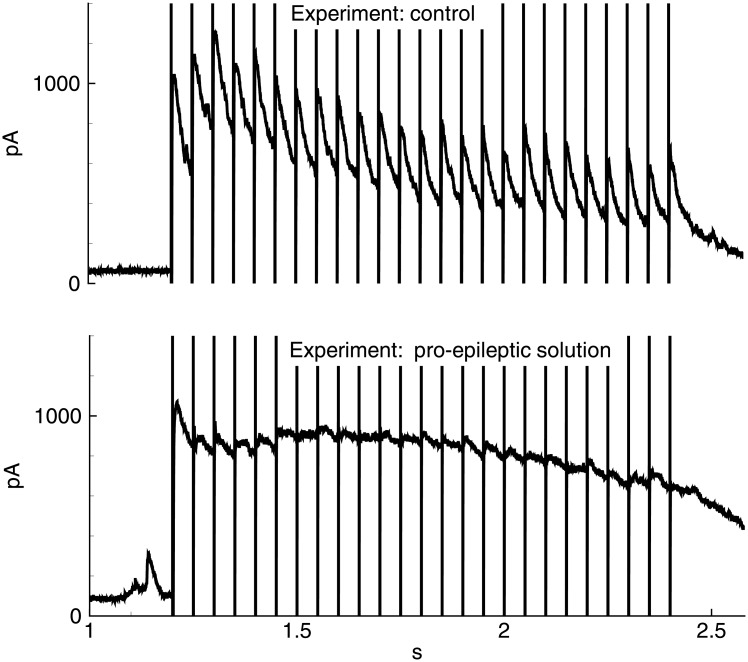
Experiment. Evoked IPSCs. IPSCs recorded at the holding voltage 0 mV in the control solution (top) and pro-epileptic solution (bottom) in response to stimulation by a train of 25 pulses of extracellular stimulation at 20 Hz.

**Fig 14:**
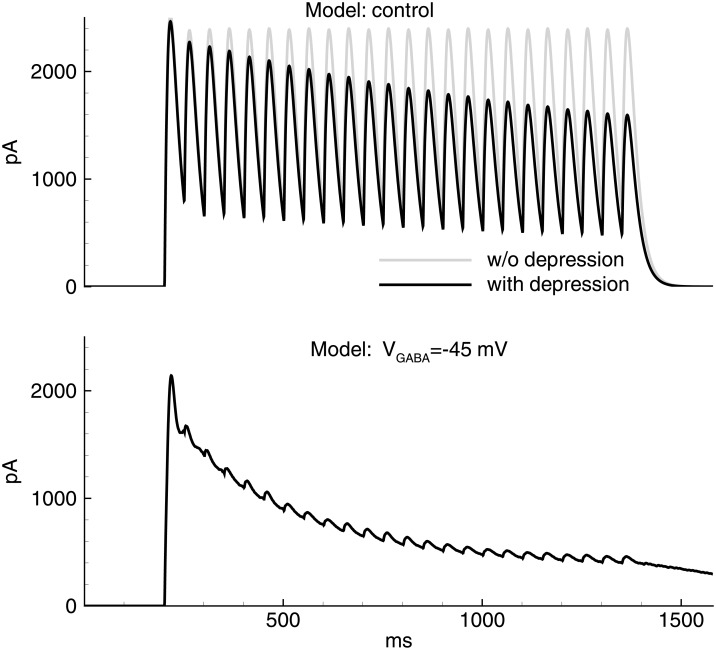
Model. Evoked IPSCs. IPSCs recorded at the holding voltage 0 mV in the control conditions with *V*_*GABA*_ = −70mV (top) and pro-epileptic conditions with *V*_*GABA*_ = −45mV (bottom) in response to stimulation by a train of 25 pulses of ‘extracellular’ stimulation at 20 Hz, as in the experiment shown in Fig 13.

Next, the role of short-term synaptic depression in discharge termination was investigated experimentally and by mathematical modelling. Isolated IPSCs evoked by a train of 25 “extracellular” pulses at 20 Hz in the model was compared with the recordings in pyramidal neurons (Figs 13 and 14). In the Ringer’s solution at the reversal potential of glutamatergic currents, an initial increase of the IPSC amplitude (first 3–5 responses within a train, Fig 13, top trace) was observed, presumably reflecting synaptic integration and/or facilitation, which was then followed by a slow decrease, indicating a short-term synaptic depression. The evoked IPSCs were monosynaptic because they exhibited a fast rise and a monotone decay.

In simulation (*V*_*GABA*_ = −70mV), a set of IPSCs with gradually decreasing amplitudes (Fig 14, top black trace) was observed. The modeled responses had a faster decay than real IPSCs. Thus, the synaptic integration did not evince. The decrease in the peaks throughout the train of IPSCs was due to synaptic depression, as determined based on the comparison to the trace obtained with the disabled synaptic depression (gray trace). The characteristic time of the decrease of the IPSC amplitudes in the train was similar to the experiment.

When the pro-epileptic solution was used in the experiment (Fig 13, bottom trace) or pro-epileptic conditions were applied in the simulation, the evoked IPSC trains were significantly different from those obtained under the control conditions (Fig 14, bottom trace). The first peak in the train exhibited a fast rise and slow decay and was followed by poorly distinguishable peaks. These changes in the shape of responses are explained by the contribution of a recurrent GABAergic current, which was absent in the control conditions. The residual difference in the slow phase of the response in the model and the experiment is presumably explained by the contribution of the GABA-B synaptic component [49], [50], which has not been modeled. The consistency between the model and the experiment was obtained only if the synaptic depression was taken into account.

**Fig 15:**
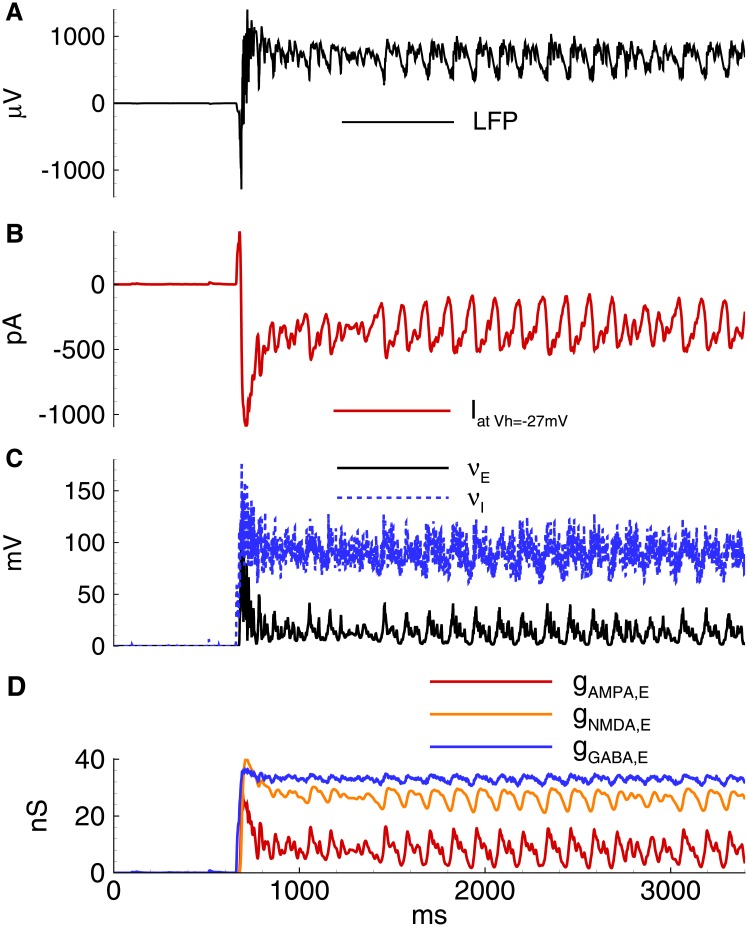
Model. Synaptic depression is necessary for the intermittency of high activity states. Model without synaptic depression switches to a continuous high activity state in the pro-epileptic condition with *V*_*GABA*_ = −45 mV. A: Local field potential (LFP). B: the membrane current at *V*_*hold*_ = −27mV; C: the firing rates for the excitatory neurons and interneurons. D: the synaptic conductances.

In a simulation with the absence of synaptic depression, the neuronal populations spontaneously switched to a high-activity state at about 650 ms (Fig 15). In contrast to the regime with IIDs, this state continued without termination. According to the model, the synaptic depression of glutamatergic synapses does not significantly change the activity in this simulation. Thus, the proposed model predicts that in the absence of the short-term depression of GABAergic synapses, the network would have a qualitatively different regime of activity in the pro-epileptic solution used.

## 4 Discussion

In this combined experimental and modeling study, a simple model was developed that reproduces experimental observations for two types of IIDs in the pyramidal neurons of the rat entorhinal cortex as well as estimations of synaptic conductances during IIDs. The model of coupled excitatory and inhibitory populations includes AMPA, NMDA, and GABA-A-receptor-mediated synapses and gap junctions. Both types of IIDs were successfully reproduced in the model by setting two different depolarized levels for GABA-mediated current reversal potential. It was revealed that short-term synaptic depression is a crucial factor for the duration and cessation of IIDs.

### 4.1 Ionic dynamics

Transmembrane gradients of ionic concentrations can be affected by ionic fluxes through channels during periods of elevated neural activity. An epileptic seizure is an example of a severely perturbed neural activity, which is accompanied by pronounced changes in intracellular and extracellular ion concentrations [48]. The ionic dynamic was not explicitly modeled in our work. Instead, the effect of chloride accumulation was taken into account in the form of *V*_*GABA*_ depolarization. This approach is in contrast to very detailed consideration of ionic dynamics in the works of Kager et al. [51], [52], [53], [54]. Their consideration proposes a detailed description of ionic dynamics for a single multi-compartmental neuron surrounded by extracellular medium and glia. The most important ionic changes are those of extracellular potassium, and intraneuronal sodium and calcium. The fast changes in ionic concentrations are mainly caused by the intrinsic voltage-gated and leak currents of a neuron, and synaptic NMDA- and GABA-mediated synaptic currents. Slower changes in ionic concentrations are mediated by Na^+^/K^+^ pump, glial uptake of potassium, Ca-buffering and Na-Ca exchanger [53].

The modeling approach for the network including inhibitory neurons and, consequently, intracellular chloride dynamics, though with some reductions of neuronal structure and ionic transport mechanisms is further developed in the several studies [26], [55], [56], [57], [58], [59]. These modeling studies together with experimental data for sodium [60], chloride [41], and potassium [19], [40], [12] show that all three ions increase their concentrations after a single IID by less than 2mM, which further relaxes during an interdischarge interval. These changes lead to the changes of reversal potentials less than 3mV. These fluctuations of the reversal potentials do not effectively change the driving force for ionic transport through channels. At the same time, the tonic elevation of the intracellular chloride and extracellular potassium concentrations are crucial factors, which can be taken into account through *V*_*GABA*_ and tonic depolarizing current, correspondingly.

Consideration of calcium dynamics shows that significant factors affecting calcium concentration are the flux through voltage-gated channels and intracellular buffering [57], whereas the main effect is on the calcium-dependent potassium channels. Similar to modeling from [34], this effect has been taken into account in our model by including the AHP-current (Section 2.2).

The observations mentioned above validate our reducing assumption about constant reversal potentials and allows us to avoid direct modeling of the ionic dynamics. We note, however, that the consideration is not valid, for example, for ictal mode or transitions between regimes.

### 4.2 GABA reversal potential

For the proposed model, it was assumed that the reversal potential of the GABAergic current is a critical factor that governs epileptic activity. The reversal potential of GABAergic currents in neurons of human and rat epileptic brains is depolarized from rest towards the action potential threshold [44], [13], [45], [18], [46]. The main factors that result in the depolarized GABA reversal potential are the chloride flux via GABAergic synapses, the downregulation and reversed operation of potassium chloride cotransporter 2 (KCC2) and voltage-gated chloride channels (ClC) [45], [61]. Sodium- and potassium-coupled chloride transport (NKCC1) may contribute to the up- and downregulations of the chloride gradient [44], [45]. Concentration changes of bicarbonate ions via Cl-/HCO3- anion exchangers, such as AE3 [62], could significantly affect the chloride extrusion rate or directly affect the GABA reversal potential via bicarbonate concentration. Another factor is the modulation of GABA transporter 1 (GAT1), which depends on the chloride concentration [63], [64]. It is hypothesized that under the conditions of a high-potassium solution and thus a suppressed KCC2 transporter, the chloride is accumulated inside neurons and leads to the suppression of the GABA uptake by GAT1, which increases the extracellular GABA concentration and thus tonically affects synaptic or extrasynaptic GABA receptors [65]. Increasing the GABA stimulation provides additional chloride flux through GABARs. The chloride accumulation that occurs due to all the mentioned factors equilibrates intra- and extracellular chloride concentrations and thus depolarizes *V*_*GABA*_. For the sake of simplicity, all these mechanisms were not taken into account in the model. Instead, only their cumulative effects that were expressed in the shift of *V*_*GABA*_ were considered.

The assumption regarding the shift of *V*_*GABA*_ to a more depolarized level in a pro-epileptic solution has been verified through experimental measurements using whole-cell configurations. Similar evidence has been reported by Yekhlef [12] in mouse medial entorhinal cortical slices during extracellular perfusion with the proconvulsive compound 4-AP. In experiments in which a perforated patch-clamp was used, *V*_*GABA*_ in interneurons was comparable to the limit value -45 mV [47] assumed in the simulation of this study. Moreover, using a gramicidin perforated patch, Fu and van den Pol [42] reported *V*_*GABA*_ to be about -35 mV in a subset of hippocampal interneurons. Thus, the *V*_*GABA*_ was more positive than the resting membrane potential and spike threshold in adult GABA interneurons that colocalized neuropeptide Y and somatostatin [42].

In the proposed model, the dynamics of the extracellular potassium concentration during IIDs was not taken into account, and thus the potassium current reversal potential was set to be constant, which is a strong assumption. Potassium accumulation in the extracellular space is associated with seizures [66]. Changes in extracellular potassium levels are mainly mediated via KCC2, which may increase neuronal excitability and contribute to seizure generation [55]. Intense neuronal firing increases extracellular potassium, further increasing neuronal excitability in a positive feedback loop that promotes seizure generation. Computational models have suggested that the changes in extracellular potassium may suffice to induce pathological conditions observed in epileptiform activity in systems of different levels, from single neurons [67], [68] to recurrent neural networks [26], [69], [56]. However, still relations between KCC2 and dynamic changes in chloride and potassium levels during the transition to seizure are not completely understood. For example, depending on the experimental conditions, KCC2 leads to pro- [59] or anti-epileptogenic effects [70]. To avoid controversy, it is suggested that the positive shift of *V*_*GABA*_ in the simulations may adequately compensate for the effect of the increase in the extracellular potassium concentration.

### 4.3 Discharge initiation by interneurons

The synchronization of a mutually interconnected network of interneurons [71], [72] occurs via recurrent collaterals as a result of depolarization mediated by synaptically activated GABAA receptors as well as electrical coupling between the interneurons [73], [74], [49].

Interictal synchronization is further facilitated by non-synaptic interactions, which can be mediated by extracellular electric fields (ephaptic interactions) or by intercellular gap junctions [75], [76], [77] that exist between either principal neurons or interneurons [78], [73], [74].

One possible mechanism for GABA discharges and waves in the mature cortex may be the elevation of extracellular potassium ions, leading to intracellular chloride accumulation due to the influx of chloride via potassium-coupled chloride transporters [71], [79], [80]. This consideration is consistent with the proposed model of IID1s and explains the initiation of IID2s.

As found by Huberfeld [10] in similar experimental conditions of high-potassium and low-magnesium concentrations but with human brain slices, interneuron firing begins the interictal events that depend on both glutamatergic and depolarizing GABAergic transmission. These interictal events appear to be similar to IID2. The prominent role of interneurons in seizure initiation has also been revealed in an optogenetic study [81]. As reported, the seizure-related interneuron activity precedes the firing of excitatory neurons. Therefore, an optogenetic inhibition of a subtype of interneurons may disrupt seizure initiation and maintenance.

A significant contribution of GABAergic conductance, similar to that during IIDs in the proposed model, was found during sharp wave-ripples [82]. It was observed that inhibition dominates excitation during the events in the hippocampal CA1 region and that phasic inhibition, though not excitation, is phase-locked to individual ripple cycles.

An alternative explanation of interneuron-based epileptic discharges was proposed by Yekhlef et al. [12], who also observed pure GABAergic preictal discharges. According to them, the depolarizing drive is attributed to a transient accumulation of extracellular potassium-mediated by KCC2 in response to the increased intracellular Cl- concentration driven by a massive activation of GABAA receptors. The authors hypothesized that interneuronal activation elicits discharges by inducing a critical increase in extracellular potassium concentration. A complementary mechanism of GABA-mediated excitation of interneurons in the entorhinal cortex was hypothesized by Uva et al. [83], who proposed that GABA-mediated chloride flux leads to a local elevation of extracellular potassium and consequently to the depolarization of interneurons, supporting the generation of ectopic, non-synaptic firing.

It has been noted that these mechanisms cannot be applied to IIDs due to the relatively slow potassium transport by means of the K-Cl cotransporters in comparison with the duration of a single IID. The potassium concentration cannot be increased by KCC2 fast enough to sustain the excitation of interneurons.

### 4.4 Role of synaptic depression

It has been shown that the transient depression of excitatory synapses on interneurons contributes to epileptiform bursts [84]. Synaptic depression [26] or AHP-currents [3] were considered in previous works as potential mechanisms of discharge termination. The data on IID1s collected in this study indicate that the synchronous discharges originate from the network of interneurons. Because the adaptation is usually not significantly prominent for at least a large fraction of interneurons [24], the AHP-based mechanism is not a likely explanation for IID termination. Alternatively, and similar to the study by Bazhenov et al. [26], this model suggests that short-term synaptic depression plays a major role. In addition to the results obtained by Bazhenov et al. [26], IIDs have not only been reproduced, but the main mechanism of discharge termination has also been revealed. The simulations suggest that the short-term synaptic depression is a key factor in IID termination. For IID1s, this conclusion is consistent with the findings by Karlocai et al. [4], who observed a strong short-term depression in the parvalbumin-positive basket cell to pyramidal cell transmission. For IID2s, it is consistent with [2].

Overall, the study has revealed the mechanisms of pathological synchronization with the primary role of excitatory GABA receptors in the interneuron network.
